# Multilocus sequence type analysis of coagulase-negative staphylococci from the neonatal intensive care unit of a teaching hospital in Ghana

**DOI:** 10.11604/pamj.2025.51.59.45565

**Published:** 2025-06-27

**Authors:** Innocent Afeke, Joseph Adu-Amankwaah, Ibrahim Jamfaru, Abdul-Wahab Mawuko Hamid, Verner Ndudiri Orish, Bright Agbodzi, Kokou Hefoume Amegan-Aho, Lennox Mac Ankrah, Hintermann Kobina Mbroh, Graceful Lord Mensah, Anthony Samuel Ablordey

**Affiliations:** 1 Department of Medical Laboratory Sciences, School of Allied Health Sciences, University of Health and Allied Sciences, Ho, Ghana,; 2 Department of Physiology, Xuzhou Medical University, Xuzhou, Jiangsu, China,; 3 Department of Microbiology and Immunology, School of Medicine, University of Health, and Allied Sciences, Ho, Ghana,; 4 Department of Bacteriology, Noguchi Memorial Institute for Medical Research, University of Ghana, Accra, Ghana,; 5 Department of Pediatrics, School of Medicine, University of Health and Allied Sciences, Ho, Ghana,; 6 Department of Obstetrics and Gynaecology, Ho Teaching Hospital, Ho, Ghana,; 7 Department of Pediatrics, Ho Teaching Hospital, Ho, Ghana

**Keywords:** Coagulase-negative staphylococci, neonatal sepsis, multilocus sequence analysis, microbial genomics, antimicrobial resistance

## Abstract

**Introduction:**

coagulase-negative staphylococci (CoNS) have emerged as significant pathogens in neonatal intensive care units (NICUs), especially as a leading cause of neonatal sepsis. In low-income countries, efforts to prevent sepsis-related mortality are hampered by a lack of comprehensive molecular data on bacterial isolates responsible for these infections. This study aims to address this gap by generating whole-genome sequencing data, identifying novel multilocus sequence (MLS) types, and investigating antibiotic resistance genes in Staphylococcus epidermidis and Staphylococcus haemolyticus from the NICU of Ho Teaching Hospital in Ghana.

**Methods:**

a total of 123 CoNS isolates were tested for minimum inhibitory concentrations (MIC) of 13 antibiotics using VITEK2, and 16 isolates (7 S. epidermidis and 9 S. haemolyticus) were selected for further genomic analysis. Genomic DNA was extracted and sequenced using an Illumina NovaSeq 6000 platform. Draft genome sequences were assembled using MEGAHIT and annotated with Prokka. MLS types were identified using the Center for Genomic Epidemiology´s MLST tool, and antibiotic resistance genes were detected via the Comprehensive Antibiotic Resistance Database (CARD) and BlastArg-annot Nt tools.

**Results:**

draft genome sequences were established for 7 S. epidermidis and 9 S. haemolyticus isolates and deposited in public databases. Five novel MLST types were discovered: ST89, ST90, and ST91 for S. haemolyticus, and ST993 and ST994 for S. epidermidis. These novel strains exhibited multidrug resistance, with multiple antimicrobial resistance genes identified in their genomes. Three of the newly identified MLST types were isolated from the blood of neonates, while two others were found on the nasal mucosa of clinical staff and a baby cot.

**Conclusion:**

our findings highlight the presence of multidrug-resistant and novel CoNS MLST types in the NICU, suggesting an urgent need for a nationwide database on CoNS MLST types circulating in Ghana. Such data would be critical for developing informed public health policies and strategies to manage neonatal sepsis in the Region.

## Introduction

Coagulase-negative staphylococci (CoNS) have emerged as clinically significant bacteria in the neonatal intensive care unit (NICU) due to their crucial implications in neonatal sepsis. *Staphylococcus epidermidis (S. epidermidis)* and *Staphylococcus haemolyticus (S. haemolyticus)* are the most frequently isolated CoNS species from clinical cases, especially bacteremia [1]. They have mainly been isolated in catheter-related bacteremia in intensive care units, mostly in the NICU [2,3]. Staphylococci, notably CoNS, have a documented history of developing resistance to antimicrobial agents. Healthcare-associated infections (HAIs) are linked with multidrug-resistant (MDR) bacteria, which increases the risk of therapeutic failure due to the limited choice of available antibiotics. HAIs caused by CoNS also lead to an extended duration of admission, increased medical costs, permanent neonatal disabilities, and higher morbidity and mortality [4].

Epidemiological research is critical for understanding clonality, evolutionary pathways, pathogen genetic diversity, and infection transmission [5]. Clonal diversity among CoNS species varies and has received far less attention than *S. aureus*. There are several tools for identifying genomic data for epidemiologic and infection control purposes. At the most basic level, phylotyping may aid epidemiological investigations by determining the source and route of infections, tracing cross-transmission of healthcare-associated pathogens, and identifying virulent antibiotic-resistant lineages or subpopulations [6].

Multiple gene nucleic acid-based typing approaches, such as multilocus sequence typing (MLST) [7] or the use of DNA arrays [8] have been proven useful over the years in assessing epidemiological relatedness among bacterial strains, especially healthcare-associated microbial species [9]. Whole-genome MLST (wgMLST) has gradually become one of the most widely used methods for bacterial strain typing [10]. However, most infections in the clinical setting are caused by bacterial strains belonging to a relatively restricted number of lineages, especially for highly prevalent methicillin-resistant *Staphylococcus aureus* (MRSA) [11]. Multilocus sequence typing coupled with multilocus variable-number tandem-repeat was successfully used to determine the genetic relatedness of Staphylococcus in the gut and skin of preterm neonates and the breast milk of their mothers [12].

Country-specific data relating to the distribution of diverse MLST types are lacking and have not yet been distinctly reported from Low- and Middle-Income Countries (LMICs). As such, this study aimed to generate whole-genome sequence data, screen for new MLS types, and identify antibiotic-resistance genes for *S. epidermidis* and *S. haemolyticus* species cultured from the NICU of Ho Teaching Hospital in Ghana.

## Methods

**Study design and site:** a cross-sectional study was conducted at the Neonatal Intensive Care Unit (NICU) of Ho Teaching Hospital (HTH), Ghana, from March to June 2018. The NICU at HTH admits neonates with conditions such as birth asphyxia, neonatal sepsis, preterm complications, and congenital abnormalities. Ethical approval for the study was obtained from the University of Health and Allied Sciences Research Ethics Committee (UHAS-REC A.2 (1) 17-18).

**Study population and sampling strategy:** a total of 305 participants were recruited using a stratified purposive sampling approach to ensure representation across key NICU-related groups: neonates (n = 118), mothers (n = 68), clinical personnel (n = 59), including doctors and nurses, and medical and nursing students (n = 60) from the University of Health and Allied Sciences who were freshly admitted. The sample size was determined based on prior similar hospital infection studies and logistical feasibility. Participants were recruited after obtaining informed consent or parental consent for neonates.

Neonates admitted to the NICU during the study period who presented with clinical signs of sepsis were included. These signs included temperature instability (fever >38°C or hypothermia <36°C), lethargy or poor feeding, respiratory distress or apnea, tachycardia (>160 bpm) or bradycardia (<100 bpm), hypotension, poor perfusion (e.g. delayed capillary refill >3 seconds or cold extremities), irritability, or seizures. Additionally, neonates with perinatal risk factors such as prematurity (gestational age <37 weeks), low birth weight (<2500 g), and a low Apgar score (<7 at 5 minutes) were included. Those at risk due to medical interventions, such as the use of central lines or umbilical catheters, or laboratory indicators like abnormal white blood cell counts (leukocytosis or leukopenia) and elevated C-reactive protein (CRP) levels, were also eligible for enrollment. Furthermore, neonates whose mothers exhibited maternal risk factors, such as prolonged rupture of membranes (>18 hours), maternal intrapartum fever (>38°C), suspected or confirmed chorioamnionitis, or untreated urinary tract or sexually transmitted infections, were included.

Mothers were included if they were present and able to provide informed consent. Clinical staff members (doctors, nurses, and midwives) working in the NICU and newly admitted students in the medical and nursing programs were also eligible for inclusion. Neonates were excluded if they were healthy term infants with no signs of sepsis or associated maternal or perinatal risk factors. Additionally, neonates with terminal conditions where medical intervention was deemed futile and only palliative care was appropriate were excluded. If parents or guardians declined participation after receiving a full explanation of the study, the neonates were excluded. For mothers, refusal to provide informed consent also resulted in exclusion. Mothers who were severely ill (e.g. admitted to intensive care) were excluded unless a qualified guardian could provide consent on their behalf. Mothers experiencing severe postpartum psychological conditions, such as psychological distress or psychosis, were also excluded due to their inability to comprehend or consent to study procedures.

### Sample collection procedures

**Blood sample collection from neonates:** peripheral blood samples (0.5-1.5 mL) were aseptically drawn by trained clinicians using sterile venipuncture techniques. Samples were inoculated into pediatric blood culture bottles (BACTEC Peds Plus™/F, BD Diagnostics) according to Clinical and Laboratory Standards Institute (CLSI) guidelines for neonatal blood cultures (CLSI M47-A).

**Nasal swab collection from mothers, clinical staff, and students:** nasal swabs were collected using sterile cotton-tipped swabs moistened with sterile saline. The swab was gently rotated in both anterior nares of each participant for 5 seconds, following with the Centers for Disease Control (CDC) protocols for nasal specimen collection. The swabs were placed in sterile transport medium and transported to the microbiology laboratory within one hour.

**Environmental surface sampling:** environmental swabbing was performed on inanimate objects in the NICU, including incubator handles, weighing scales, and baby cots. Pre-moistened sterile swabs (moistened with sterile saline) were used to swab a 5 x 5 cm area using a rolling and rotating technique, as outlined in the World Health Organization (WHO) guidelines on environmental hygiene surveillance. The selection of objects like the baby cot and weighing scale for microbial sampling was based on their frequent and direct use in neonatal care. These surfaces are essential to daily infant monitoring and are potential reservoirs for microbial contamination. Swabbing them while actively in use provides an accurate representation of the microbial environment to which neonates are exposed, aiding in infection prevention and control in the NICU.

**Sample transport and microbiological processing:** all specimens were transported promptly to the microbiology laboratory for analysis. Blood culture bottles were transported at room temperature to maintain organism viability and optimize culture sensitivity, in line with Clinical and Laboratory Standards Institute (CLSI) guidelines. In contrast, nasal and environmental swab samples were transported in cold boxes maintained at 2-8°C to minimize overgrowth and preserve microbial integrity. Upon arrival at the laboratory, blood cultures were loaded into the BACTEC 9050 automated blood culture system (BD Diagnostics) for incubation and continuous monitoring. Positive cultures were subcultured onto 5-10% sheep blood agar (Oxoid, UK) and incubated aerobically at 37°C for 24-48 hours. Swab samples were streaked directly onto 5% sheep blood agar plates and incubated aerobically at 37°C. Plates with no visible growth at 24 hours were re-incubated for an additional 24 hours to ensure detection of slow-growing organisms. Bacterial colonies were characterized based on morphology, Gram staining, and standard biochemical tests. Isolates were preserved in 15% glycerol broth and stored at -80°C for downstream analyses.

**Bacterial culture:** a total of 527 bacterial isolates were recovered from samples collected from neonates, mothers (nasal mucosa), clinical staff, medical and nursing students, and objects. Among these, 123 coagulase-negative staphylococci (CoNS) isolates, consisting solely of *Staphylococcus haemolyticus* and *S. epidermidis*, were obtained for antimicrobial susceptibility testing (AST). Based on AST profiles, 16 isolates (9 *S. haemolyticus* and 7 *S. epidermidis*) were purposively selected for whole-genome sequencing (WGS). The selection was guided by several criteria: 1) antibiotic resistance diversity: isolates displaying varied resistance profiles, including multidrug-resistant phenotypes, were prioritized to allow comprehensive analysis of antimicrobial resistance genes and mechanisms; 2) source representation: isolates were selected to ensure diversity in sources (e.g. neonates, mothers, staff, and environment) to investigate potential transmission dynamics and colonization patterns across different reservoirs; 3) species balance: both *S. haemolyticus* and *S. epidermidis* were included to represent the predominant CoNS species identified and to compare their genomic features and resistance determinants; 4) clinical relevance: preference was given to isolates recovered from neonates and those associated with potential clinical significance, given the vulnerability of neonates to opportunistic infections. This selection strategy was designed to maximize the utility of WGS in characterizing resistance mechanisms, assessing potential horizontal gene transfer, and understanding epidemiological linkages among participants and their environment.

**Bacterial isolates:** from March to June 2018, blood samples were cultured using a BACTEC 9050 system. Positive culture bottles were subcultured on 5 to 10% sheep blood agar, collected, and kept in Brain Heart Infusion (BWI) (Oxoid Ltd., England) with 15% glycerol at -20°C for further study. Nasal mucosa swab samples from mothers, clinical staff, medical and nursing students, and inanimate objects were inoculated on 5% sheep blood agar plates and incubated aerobically at 37°C for 24 hours. Plates with no growth were re-incubated for an additional 24 hours. Colonies on blood agar plates were identified by morphology and gram staining as gram-positive cocci. A straight wire was used to transfer colonies to fresh blood agar plates for subculture. Gram-positive cocci were confirmed by a second Gram staining and preserved in Brain Heart Infusion (BWI) with 15% glycerol.

**Identification of bacterial isolates:** all of the bacterial isolates were identified using the MALDI-TOF Biotyper® (Bruker Daltonik, Massachusetts, USA) at the University of Lübeck's, Department of Infectious Diseases and Microbiology, as described previously [13,14]. For CoNS, the *tuf* gene provides a reference method with high accuracy for recognizing hospital infections related to *S. epidermidis* and *S. haemolyticus* [15,16]; therefore, the results of MALDI-TOF were validated using *tuf* gene sequence typing as demonstrated by Hwang *et al*. [17]. Briefly, polymerase chain reaction (PCR) amplification of the *tuf* gene was carried out on a C1000 Touch™ Thermal Cycler (BioRad) by using a set of primers 5´-GCCAGTTGAGGACGTATTCT-3' and 5´-CCATTTCAGTACCTTCTGGTAA-3', which amplify a 412 bp fragment of the *tuf* gene. The PCR products were aliquoted (46 μl) into 1 ml Eppendorf tubes, sealed, and transported to GENEWIZ-Brooks Life Sciences, Leipzig, Germany, for *tuf* gene sequencing. DNA sequencing was performed by the Sanger method. The *tuf* gene sequences were aligned independently for each isolate using MEGA 5 (Molecular Evolutionary Genetics Analysis) software and compared to all available CoNS sequences listed in the GenBank database.

**Antimicrobial susceptibility testing:** overnight cultured bacterial isolates on blood agar media were examined for purity, and 0.5 McFarland concentrations were prepared with the manufacturer's diluent and tested with a bench-top turbidimeter. The inoculum was added to a VITEK 2 AST card per the manufacturer's instructions. The reagent card was inserted into the machine for analysis. A purity check plate was carried out by plating the diluted suspension on blood agar, incubated aerobically at 37°C overnight. The MICs of 17 antibiotics were ascertained for these strains by VITEK 2® machine (bioMérieux, Durham, USA). *S. aureus* NCTC 12493 was included as a susceptible quality control strain. The MIC results were interpreted according to the 2018 European Committee on Antimicrobial Susceptibility Testing (EUCAST) clinical breakpoints. The MICs of mupirocin, teicoplanin, vancomycin, and tobramycin were also tested using gradient strips (E-test®; Liofilchem® s.r.l., Italy) using Mueller-Hinton E agar (bioMérieux SA, Strasbourg, France).

**Genomic DNA extraction:** the DNeasy blood and tissue kit (Germany-based Qiagen) was used to extract genomic DNA. The Gram-positive bacteria extraction protocol provided in the kits was adhered to without alteration.

**Whole-genome sequencing and genome annotation:** the whole-genome sequencing was carried out by Novogene (UK) Ltd. The NEBNext Ultra DNA library preparation kit (New England Biolabs, Ipswich, MA, USA) was used to prepare the library according to the manufacturer's instructions. A detailed sequencing procedure has been previously described [14]. MEGAHIT v1.2.9(5) was used to assemble sequencing reads. Gene prediction and functional annotation were done using Prokka v1.14.6(6) with the BLAST, Pfam, and NCBI databases. The aforementioned software package was used with default parameters. The whole-genome sequence data of the isolates have been deposited in GenBank with accession numbers, as shown in Annex 1.

**Bioinformatics analyses:** a multilocus sequence typing (MLST) online search was carried out as described previously [18]. Antibiotic resistance genes were searched for with the CARD Resistance Gene Identifier [19] and BlastArg-annot Nt websites. The staphylococcal cassette chromosome mec (SCCmec) type was ascertained with SCCmec Finder v1.2 [20].

**Ethics approval and consent to participate:** the study was conducted in accordance with the Declaration of Helsinki. The Research Ethics Committee of the University of Health and Allied Sciences (UHAS), Ghana, reviewed and approved this study with Protocol Identification Number UHAS-REC/A.2(1)17-18. Written approval was also obtained from the Ho Teaching Hospital (HTH) to use the facility for the study. Informed consent was obtained from all subjects involved in the study.

## Results

**Multilocus Sequence Typing (MLST) reveals novel and predominant sequence types of *S. epidermidis* and *S. haemolyticus* isolates from neonates, mothers, and clinical staff:** for the *S. epidermidis* species, of which multilocus sequence types were analyzed, two of the five isolates (2/5) cultivated from the neonates' blood samples have MLS type 490. Two isolates (2/5) had no match in the MLS database and were assigned new MLS types 993 and 994. One mother's isolate had MLS type 48 (Table 1).

**Table 1:** molecular characterization of selected *S. epidermidis* and *S. haemolyticus* based on their multilocus sequence types

*Staphylococcus epidermidis*	Sample ID	MLS type	MLS type assigned
	^c^HESN035b	48	NA
	^c^HESS022*	Unknown	None
	^i^HESN038B	Unknown	994
	^i^HESN090B	490	NA
	^i^HESN074B	Unknown	993
	^i^HESN016B	490	NA
	^i^HESN103B	226	NA
***Staphylococcus Haemolyticus***			
	^i^HESN036B	1	NA
	^i^HESN094B	1	NA
	^c^HESN035a	30	NA
	^c^HESN072a	3	NA
	^c^HESMS053a	1	NA
	^i^BABY089B^β^	Unknown	90
	^o^MW015	Unknown	91
	^c^HESMS017b	Unknown	89
	^i^BABY162B^β^	1	NA

MLS: multilocus sequence; NA: not applicable

For *S. haemolyticus* isolates, three of the four isolates (3/4) cultivated from neonates' blood samples had MLS type 1, and the other one had no match in the database, although it was close to MLS type 30 as indicated by the search tool (Table 1). Nasal mucosae isolates from two clinical staff had MLS type 1 and an unknown MLS (close to MLS type 60). Two of the mothers' isolates analyzed had MLS types 3 and 30. All unknown MLS (miscellaneous data) types were assigned novel MLS types except one, as highlighted in Table 1. One of the unknown *S. epidermidis* isolates was not assigned because it had a novel MLS type with a missing sequence for one of the housekeeping genes (yqiL).

**Figure 1:**
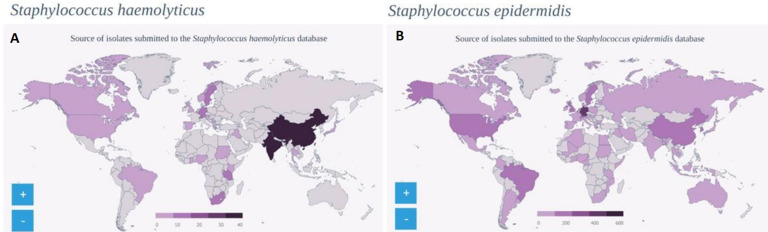
coagulase-negative staphylococci (CoNS) multilocus sequence type database A): *Staphylococcus haemolyticus* B): *Staphylococcus epidermidis*

A check from the global database of new MLS types submitted from various geographic areas revealed that the three new MLS types identified in this study for *S. haemolyticus* were the only submissions from Ghana (Figure 1A). The two new MLS types of *S. epidermidis* reported in this study were two of three submitted to the database from Ghana (Figure 1B).

**Antimicrobial susceptibility patterns reveal high resistance rates in *S. haemolyticus* and variable resistance in *S. epidermidis* isolates:** for *S. epidermidis*, using the results of cefoxitin and oxacillin to define methicillin resistance, only one of the seven isolates was methicillin-resistant. However, two of the seven isolates showed discordant results for these two antibiotics, with one isolate having no reported cefoxitin results. The *S. epidermidis* isolates showed the highest resistance to tetracycline (5/7), followed by clindamycin and gentamicin, each of three isolates out of the seven. No resistance was observed for the following antibiotics: tigecycline, linezolid, and vancomycin (Table 2).

**Table 2:** antimicrobial susceptibility testing for *Staphylococcus epidermidis*

ID	CEF	OXA	ERY	CL	GEN	TET	TIG	CIP	MOX	RIF	TEI	VAN	MUP	FOS
^c^HESNO35a	2[S]	0.25[S]	0.25[S]	0.25[S]	0.5[S]	>16[R]	ND	ND	0.25[S]	0.5[S]	2[S]	1[S]	ND	8[S]
^c^HESS022	>8[R]	2[S]	0.25[S]	0.25[S]	0.5[S]	>16[R]	0.12[S]	0.12[S]	0.25[S]	0.5[S]	8[R]	1[S]	ND	>128[R]
^i^HESN038B	2[S]	0.25[S]	0.25[S]	0.25[S]	1[S]	0.5[S]	0.12[S]	0.25[S]	0.25[S]	0.25[S]	1[S]	1[S]	1[S]	16[S]
^i^HESNO90B	ND	>2[R]	>4[R]	>8[R]	>4[R]	>16[R]	0.5[S]	>4[R]	ND	0.25[S]	2[S]	1[S]	1[S]	16[S]
^i^HESN074B	2[S]	2[S]	0.5[S]	0.25[S]	>4[R]	0.5[S]	ND	0.25[S]	0.25[S]	0.25[S]	1[S]	0.5[S]	1[S]	16[S]
^i^HESN016B	2[S]	>2[R]	0.5[S]	>8[R]	2[S]	>16[R]	0.12[S]	2[S]	0.25[S]	>32[R]	1[S]	0.5[S]	>256[R]	>64[R]
^i^HESN103B	>8[R]	>2[R]	>4[R]	>8[R]	>4[R]	>16[R]	0.12[S]	4[S]	ND	0.25[S]	1[S]	1[S]	1[S]	16[S]

In the case of *S. haemolyticus*, apart from one isolate for which cefoxitin was not reported, there was an agreement between the results of the two antibiotics used to define methicillin resistance (Table 3). Our study revealed 100% (9/9) methicillin resistance among the nine *S. haemolyticus* isolates selected. The antibiotics erythromycin and tetracycline were identified to have the highest resistance for this Staphylococcus species, each of the seven isolates out of the nine isolates analyzed. Gentamicin, a broad-spectrum antibiotic used among antibiotics to treat infants in the Ho Hospital, was the second (6/9) antibiotic that showed resistance among the *S. haemolyticus* isolates tested. No resistance was observed against the following antibiotics: vancomycin, linezolid, and daptomycin. However, resistance was observed for two antibiotics, tigecycline (5/9) and fosfomycin (4/8), classified by WHO Essential Medicines List Working Group as reserved antibiotics, which are last-resort antibiotics for targeted use in multidrug-resistant infections.

**Table 3:** antimicrobial susceptibility testing for *Staphylococcus haemolyticus*

ID	CEF	OXA	ERY	CL	GEN	TET	TIG	CIP	MOX	RIF	TEI	VAN	MUP	FOS
^i^HESN036B	>8[R]	>4[R]	>8[R]	>8[R]	4[R]	>16[R]	0.5[S]	>4[R]	1[R]	0.25[S]	2[S]	1[S]	>256[R]	32[S]
^i^HESN094B	>8[R]	>4[R]	0.25[S]	0.25[S]	4[R]	2[S]	0.12[S]	>4[R]	0.25[S]	0.25[S]	2[S]	0.5[S]	1[S]	32[S]
^c^HESN035b	ND	>4[R]	>4[R]	0.25[S]	4[R]	>16[R]	1[R]	ND	1[R]	0.5[S]	4[S]	1[S]	ND	>128[R]
^c^HESN072a	>8[R]	>4[R]	0.25[S]	0.25[S]	4[R]	>16[R]	1[R]	ND	0.25[S]	>32[R]	2[S]	1[S]	ND	>128[R]
^c^HESMS053a	>8[R]	>4[R]	>8[R]	>8[R]	>16[R]	>16[R]	1[R]	>4[R]	>8[R]	>32[R]	4[S]	1[S]	1[S]	64[S]
^i^BABY089B	>8[R]	>4[R]	>8[R]	>8[R]	0.5[S]	>16[R]	1[R]	ND	0.25[S]	>32[R]	2[S]	0.5[S]	ND	>128[R]
^o^MW015	>8[R]	>4[R]	>8[R]	0.25[S]	0.5[S]	>16[R]	>2[R]	ND	0.25[S]	0.5[S]	2[S]	0.5[S]	ND	>128[R]
^c^HESMS017b	>8[R]	>4[R]	>8[R]	0.5[S]	2[S]	0.5[S]	0.12[S]	0.5[S]	0.5[S]	0.5[S]	1[S]	2[S]	>256[R]	ND
BABY162B	>8[R]	>2[R]	>8[R]	0.25[S]	>4[R]	>16[R]	0.5[S]	>4[R]	ND	>32[R]	>4[R]	2[S]	1[S]	16[S]

**Molecular characterization reveals variable antimicrobial resistance gene profiles and limited biofilm-forming potential among *S. epidermidis* and *S. haemolyticus isolates*:** the seven *S. epidermidis* isolates have a similar genomic size of around 2 x 10^6^ and GC contents of either 31 or 32%. SCCmec subtypes were identified in three of the seven (3/7) isolates' genomes; biofilm-forming genes and icaBCD genes were detected on the genomes of three of the seven (3/7) isolates. The seven isolates harbored varying numbers of antimicrobial-resistant genes, with one isolate having the highest number of eleven genes and another having at least five. The *mec*A gene was identified in only one isolate (Table 4). Genomic analysis of data revealed that all nine *S. haemolyticus* isolates have similar genomic sizes of around 2 x 10^6^ and GC contents of about 32%. SCCmec sub-types were identified on the genomes of two of the nine (2/9) isolates. None of the nine *S. haemolyticus* isolates harbored the biofilm-forming genes (Table 5). Most (4/9) of these Staphylococcal species harbored nine different antimicrobial-resistant genes, while an isolate harbored at least three. Although the antimicrobial susceptibility testing results for the nine isolates suggest they are methicillin-resistant, only three harbored the *mec*A gene on their genomes.

**Table 4:** molecular characterization and antimicrobial resistance gene carriage of *Staphylococcus epidermidis*

ID	Genome size	Contigs	GC content %	CDS	rRNA	tRNA	tmRNA	SCCmec type	Antimicrobial resistance gene	Biofilm gene
^c^HESNO35a	2374923	55	31.96	2195	4	33	1	None	APH(3'),blaZ, dfrC, far1, fusB, norA, & IS1272	icaB,C,D
^c^HESS022	2919991	276	31.74	2798	5	39	1	None	APH(3'), blaI, blaR1, blaZ, dfrC, & IS1272	None
^i^HESN038B	2682678	115	31.8	2508	8	51	0	None	APH(3'), dfrC, norA, gyrB, IS1272	None
^i^HESNO90B	2417530	41	32.09	2256	6	51	1	IVa (2B)	APH(3'), blaI, blaR1, blaZ, dfrC, dfrG, dfrK, tet(K), norA, gyrB & IS1272	icaB,C,D
^i^HESN074B	2439254	30	32.01	2238	8	52	1	Vc(5c2&5)	APH(3'), dfrC, far1, fusB, norA, gyrB & mecA	None
^i^HESN016B	2411715	22	32.11	2241	6	53	1	Vc(5c2&5)	APH(3'), blaI, blaR1, blaZ dfrC, dfrG, dfrK, norA, gyrB & IS1272	icaB,C,D
^i^HESN103B	2431156	25	32	2233	8	49	1	None	APH(3'), blaI, blaR1, blaZ, dfrC, dfrG, dfrK, norA, gyrB & IS1272	None

**Table 5:** molecular characterization and antimicrobial resistance gene carriage of *Staphylococcus haemolyticus*

ID	Genome size bp	Contigs	GC content %	CDS	rRNA	tRNA	tmRAN	SCCmec type	Antimicrobial resistance gene	Biofilm gene
^i^HESN036B	2557090	67	32.55	2526	6	58	1	Vc(5c2&5)	APH(3'), blaI, blaR1, blaZ, dfrG, dfrK, mecA & IS1272	None
^i^HESN094B	2630890	90	32.49	2568	6	49	1	Vc(5c2&5)	APH(3'), blaI, blaR1, blaZ, dfrG, dfrK, mecA & IS1272	None
^c^HESN035b	2660314	170	32.74	2569	5	80	1	None	APH(3'), blaI, blaR1, blaZ, dfrG, dfrK, tet(M), tet(S), mphC, msrA & IS1272	None
^c^HESN072a	2758447	114	32.5	2717	6	59	1	None	APH(3'), blaI, blaR1, blaZ, dfrG, dfrK, , far1, ??fusB & IS1272	None
^c^HESMS053a	2396859	69	32.66	2334	6	61	1	None	APH(3'), blaI, blaR1, blaZ, dfrG, dfrK, , mphC, msrA & IS1272	None
^i^BABY089B	2453213	69	32.75	2404	7	44	1	None	APH(3'), blaI, blaR1, blaZ, dfrG, dfrK, tet(45), tet(L)& IS1272	None
^o^MW015	2873520	190	32.6	2809	7	58	1	None	APH(3'), dfrG, dfrK, msrA, mecA	None
^c^HESMS017b	2462495	20	32.78	2382	5	57	1	None	APH(3'), NorA & fosB	None
^i^BABY162B	2397901	66	32.64	2337	6	51	1	None	APH(3'), blaI, blaR1, blaZ, dfrG, dfrK, mphC, msrA & IS1272	None

## Discussion

Current data reveal a limited number of new MLS types of *S. epidermidis* and *S. haemolyticus* submitted to the Global Database from West Africa. Surprisingly, only a few countries, such as Mali, Nigeria, and Ghana, have submitted data for *S. epidermidis*. In the case of *S. haemolyticus*, which has a globally limited number of new MLS reports, only Nigeria (from West Africa) had submitted data, and the new MLS report in our study was from Ghana. This raises the question of why there is such a scarcity of new MLS types from these two staphylococcal species in West Africa. Meanwhile, a few studies have reported that understanding the population structure of bacterial species is vital for epidemiology and public health intervention, as well as for addressing more fundamental questions concerning the evolution, persistence, and adaptation of that species [21]. Interestingly, plotting the provenance of an isolate on a geographical map and relating it to its genotypic placement in a phylogenetic tree can be helpful when investigating the structuring of outbreaks or the global spread of clones [21]. *S. epidermidis* and *S. haemolyticus* are the most commonly isolated CoNS species from clinical cases, including bacteremia and sepsis, especially in patients with in-plant devices and catheter-related bacteremia, mostly in intensive care units and NICUs [1]. Unfortunately, most sub-Saharan African countries experience high morbidity and mortality due to bloodstream infections from these two CoNS species in the NICUs [22]. Hence, there is an urgent need for healthcare facility-based, local, national, and global MLS databases for these two staphylococcal species to inform healthcare policies.

In our study, we identified five novel MLS types from sixteen sequenced CoNS isolates. These new MLS types were found in different species of Staphylococci, including *S. epidermidis* and *S. haemolyticus*. Interestingly, two new MLS types were found in *S. epidermidis* isolates from blood samples of neonates and young infants at the Ho Teaching Hospital in Ghana, where our study was conducted. The other three isolates were *S. haemolyticus* species, each cultured from a neonate's blood sample, the clinical staff's nasal mucosa, and a swab from a baby's cot. Notably, four of the nine *S. haemolyticus* isolates sequenced were ST 1 and cultured from neonates' blood samples (three) and the nasal mucosa of clinical staff. Moreover, we discovered that two *S. haemolyticus* isolates, cultured from the nasal mucosae of two mothers, had different MLS types (ST 3 and ST 30), indicating that ST 1 may be an endemic strain causing bloodstream infections among the neonates. Better still, the isolates from the two mothers had different STs, suggesting the possibility of heterogeneous STs among the mothers. However, it is difficult to stipulate that the five new MLS types identified in our study are hospital-based endemic strains, as we have sequenced a limited number of isolates and included only a few mothers and student isolates, which may represent community-based isolates. It is worth noting that the students in our study were first-year medical and nursing students from various regions of Ghana who were freshly admitted to the University of Health and Allied Sciences without prior contact with the Ho Teaching Hospital. Additionally, we did not randomly select the isolates for whole-genome sequencing, but rather selected isolates based on their antimicrobial resistance profile, specifically their multidrug resistance or resistance to selected antimicrobials like tigecycline and mupirocin, to predict their molecular mechanisms of resistance.

There is limited data on antibiotics' resistance profiles for CoNS, especially isolates in the NICUs of Ghanaian hospitals involved in bloodstream infections among babies. The current study determined antimicrobial susceptibility profiles for *S. epidermidis* and *S. haemolyticus* isolates cultivated from babies' blood samples, nasal mucosae of mothers, clinical staff, and students without contact with the HTH wards. Two of the *S. epidermidis* gave discordant results for cefoxitin and oxacillin MICs, making it difficult to determine their methicillin status. In this case, the interpretation depends on the individual laboratories' guidelines for interpreting discordant results of oxacillin and cefoxitin MICs. The CDC guideline for determining methicillin susceptibility suggested that, although oxacillin is more likely to detect heteroresistant strains, which makes it better than methicillin, cefoxitin is a better inducer of the mecA gene. Tests using cefoxitin give more reproducible and accurate results than tests with oxacillin [21].

Phenotypically, all five *S. haemolyticus* isolates are methicillin-resistant. However, only two of them carried the mecA gene on their genomes. This phenomenon is not common in staphylococcal species. Although mecA gene expression is usually a prerequisite for methicillin resistance, mecA expression alone does not appear to be sufficient to guarantee phenotypic methicillin resistance, suggesting additional molecular targets that could be associated with the susceptibility to oxacillin in certain strains [23]. The opposite of this occurrence is seen with the *S. epidermidis* strain HESNO74B, where the mecA gene was identified, but the isolate was phenotypically susceptible to most antibiotics. A study has investigated this phenomenon and reported that mutations in various genes might have been responsible for this observation [23]. It is interesting to note that a prior study showed that developing drug resistance to one drug significantly altered drug resistance and sensitivity to other drugs. This study suggests that the phenotypic alterations of the resistant strains were not always limited to certain causes, such as changing the structure of the drug target protein, but instead resulted in changes in a number of intracellular features [24]. A complicated interaction network is implicated in the phenomenon of drug resistance because it involves changes in a number of components, including the genome, transcripts, and metabolites [24]. Antibiotic exposure has been identified as the most important factor in the emergence and spread of antibiotic resistance [24]. This perspective highlights the role of natural (Darwinian) selection in the evolution of resistance, resulting in the survival and procreation of antibiotic-resistant species while their susceptible counterparts become extinct [25].

Identifying specific genes responsible for resistance to antibiotics helps to understand the resistance mechanism for those antibiotics. This study identified a trend of common antibiotic genes like *APH*(3') *bla*I, *bla*R1, *bla*Z, *dfr*G, and *dfr*K for both groups suggesting that, phenotypically, all the *S. haemolyticus* isolates are methicillin-resistant. A known attribute of certain CoNS species strains that makes them more pathogenic than just commensals is the production of biofilms. Biofilm formation facilitates resistance against host immunity [26] and confers antimicrobial and biocide resistance [27]. Two of the *S. epidermidis*, both cultivated from neonates' blood samples, harbored the biofilm-forming genes icaB, C, and D. Clusters of CoNS may be spread among newborns and hospital workers, according to biochemical and molecular typing investigations, while isolates linked to sepsis may be more homogeneous [28]. It has been demonstrated that single clones of multi-resistant *S. epidermidis* and *S. haemolyticus* strains that generate biofilms are connected to colonization and morbidity in premature newborns in NICUs [29]. Within a hospital, clonal expansion of endemic, multidrug-resistant CoNS was also observed in non-neonatal ICUs and wards. In a Dutch neonatal ICU, one genetic cluster emerged as the leading cause of CoNS sepsis after 11 years [30]. Furthermore, the possibility of inter-hospital dissemination has been proven [31]. Conversely, a significant genetic diversity of *S. epidermidis* was discovered in healthy, non-hospitalized individuals [32].

## Conclusion

*Staphylococcus epidermidis* and *S. haemolyticus* are ubiquitous inhabitants of the human skin and mucous membranes. However, their presence in NICUs is particularly concerning as they can cause severe infections in vulnerable newborns. Through our study, we have employed cutting-edge whole-genome sequencing techniques to gain new insights into the genomic diversity of these bacteria and identified novel multilocus sequence types (MLSTs) associated with infections in neonates in a Ghanaian hospital. Moreover, our analysis has uncovered various antibiotic-resistance genes in these isolates, highlighting the urgent need for effective infection control strategies in NICUs. These findings could be crucial in developing new treatment options for neonatal infections caused by these bacteria, ultimately improving the health outcomes of the most vulnerable patients in our hospitals.

**Limitation:** this study was limited since the whole-genome sequencing was only performed on a limited number of *S. haemolyticus* and *S. epidermidis* isolates from a specific population. This limitation highlights the need for more comprehensive studies with larger sample sizes to obtain more accurate and generalizable data. Without a larger dataset, the study's conclusions based on MLST whole-genome sequencing data cannot be extrapolated to the broader population. As such, the results should be interpreted cautiously, and further research is needed to validate the findings and better understand CoNS antimicrobial resistance and biofilm formation mechanisms in NICUs.

### What is known about this topic

- Staphylococcus epidermidis and Staphylococcus haemolyticus are the most commonly isolated CoNS species associated with clinical cases of bacteremia and sepsis in NICUs, particularly in sub-Saharan Africa, where neonatal sepsis leads to high morbidity and mortality rates;
- Understanding the population structure and genomic diversity of bacterial species is essential for epidemiological studies and the development of public health interventions aimed at controlling the spread of infections;
- The mecA gene is typically responsible for methicillin resistance in staphylococcal species, but the presence of this gene does not always correlate with phenotypic methicillin resistance, indicating more complex resistance mechanisms.

### What this study adds

- This study identified five novel MLS types of S. epidermidis and S. haemolyticus in NICU isolates from Ghana, contributing new data to the global MLS database and highlighting gaps in the molecular epidemiology of CoNS in West Africa;
- The study revealed the presence of multidrug-resistant S. haemolyticus and S. epidermidis isolates, including strains from neonates' blood samples, clinical staff, and environmental samples, underscoring the need for stringent infection control measures in NICUs;
- Biofilm-forming genes were identified in S. epidermidis isolates from neonates, which may enhance their pathogenic potential, emphasizing the role of biofilms in antimicrobial resistance and the spread of CoNS in hospital settings.

## References

[1] Michels R, Last K, Becker SL, Papan C (2021). Update on Coagulase-Negative Staphylococci-What the Clinician Should Know. Microorganisms.

[2] Eltwisy HO, Twisy HO, Hafez MH, Sayed IM, El-Mokhtar MA (2022). Clinical Infections, Antibiotic Resistance, and Pathogenesis of *Staphylococcus haemolyticus*. Microorganisms.

[3] França A (2023). The Role of Coagulase-Negative Staphylococci Biofilms on Late-Onset Sepsis: Current Challenges and Emerging Diagnostics and Therapies. Antibiotics (Basel).

[4] Serra-Burriel M, Keys M, Campillo-Artero C, Agodi A, Barchitta M, Gikas A (2020). Impact of multi-drug resistant bacteria on economic and clinical outcomes of healthcare-associated infections in adults: Systematic review and meta-analysis. PLoS One.

[5] Hsu LY, Harris SR, Chlebowicz MA, Lindsay JA, Koh TH, Krishnan P (2015). Evolutionary dynamics of methicillin-resistant Staphylococcus aureus within a healthcare system. Genome Biol.

[6] Schürch AC, Arredondo-Alonso S, Willems RJL, Goering RV (2018). Whole genome sequencing options for bacterial strain typing and epidemiologic analysis based on single nucleotide polymorphism versus gene-by-gene-based approaches. Clin Microbiol Infect.

[7] Guerrero-Araya E, Muñoz M, Rodríguez C, Paredes-Sabja D (2021). FastMLST: A Multi-core Tool for Multilocus Sequence Typing of Draft Genome Assemblies. Bioinform Biol Insights.

[8] Michael Dunne W, Pouseele H, Monecke S, Ehricht R, van Belkum A (2018). Epidemiology of transmissible diseases: Array hybridization and next generation sequencing as universal nucleic acid-mediated typing tools. Infect Genet Evol.

[9] Sabat AJ, Budimir A, Nashev D, Sá-Leão R, van Dijl J, Laurent F (2013). Overview of molecular typing methods for outbreak detection and epidemiological surveillance. Euro Surveill.

[10] Liu YY, Lin JW, Chen CC (2019). cano-wgMLST_BacCompare: A Bacterial Genome Analysis Platform for Epidemiological Investigation and Comparative Genomic Analysis. Front Microbiol.

[11] Said KB, Alghasab NS, Alharbi MSM, Alsolami A, Saleem M, Alhallabi SA (2023). Molecular and Source-Specific Profiling of Hospital *Staphylococcus aureus* Reveal Dominance of Skin Infection and Age-Specific Selections in Pediatrics and Geriatrics. Microorganisms.

[12] Soeorg H, Metsvaht HK, Keränen EE, Eelmäe I, Merila M, Ilmoja ML (2019). Genetic Relatedness of Staphylococcus haemolyticus in Gut and Skin of Preterm Neonates and Breast Milk of Their Mothers. Pediatr Infect Dis J.

[13] Afeke I, Hirose M, Amegan-Aho KH, Haertel C, Becker M, Moustafa A (2021). Neonatal and Young Infant Sepsis in a Regional Hospital in Ghana. Open Journal of Pediatrics.

[14] Afeke I, Moustafa A, Hirose M, Becker M, Busch H, Kuenstner A (2021). Draft Genome Sequences and Antimicrobial Profiles of Three Staphylococcus epidermidis Strains from Neonatal Blood Samples. Microbiol Resour Announc.

[15] Alexopoulou K, Foka A, Petinaki E, Jelastopulu E, Dimitracopoulos G, Spiliopoulou I (2006). Comparison of two commercial methods with PCR restriction fragment length polymorphism of the tuf gene in the identification of coagulase-negative staphylococci. Lett Appl Microbiol.

[16] Capurro A, Artursson K, Waller KP, Bengtsson B, Ericsson-Unnerstad H, Aspán A (2009). Comparison of a commercialized phenotyping system, antimicrobial susceptibility testing, and tuf gene sequence-based genotyping for species-level identification of coagulase-negative staphylococci isolated from cases of bovine mastitis. Vet Microbiol.

[17] Hwang SM, Kim MS, Park KU, Song J, Kim EC (2011). Tuf gene sequence analysis has greater discriminatory power than 16S rRNA sequence analysis in identification of clinical isolates of coagulase-negative staphylococci. J Clin Microbiol.

[18] Larsen MV, Cosentino S, Rasmussen S, Friis C, Hasman H, Marvig RL (2012). Multilocus sequence typing of total-genome-sequenced bacteria. J Clin Microbiol.

[19] Alcock BP, Raphenya AR, Lau TTY, Tsang KK, Bouchard M, Edalatmand A (2020). CARD 2020: antibiotic resistome surveillance with the comprehensive antibiotic resistance database. Nucleic Acids Res.

[20] Kaya H, Hasman H, Larsen J, Stegger M, Johannesen TB, Allesøe RL (2018). SCCmecFinder, a Web-Based Tool for Typing of Staphylococcal Cassette Chromosome mec in Staphylococcus aureus Using Whole-Genome Sequence Data. mSphere.

[21] Jolley KA, Bray JE, Maiden MCJ (2018). Open-access bacterial population genomics: BIGSdb software, the PubMLST.org website and their applications. Wellcome Open Res.

[22] Seale AC, Obiero CW, Jones KD, Barsosio HC, Thitiri J, Ngari M (2017). Should First-line Empiric Treatment Strategies for Neonates Cover Coagulase-negative Staphylococcal Infections in Kenya?. Pediatr Infect Dis J.

[23] Boonsiri T, Watanabe S, Tan XE, Thitiananpakorn K, Narimatsu R, Sasaki K (2020). Identification and characterization of mutations responsible for the β-lactam resistance in oxacillin-susceptible mecA-positive Staphylococcus aureus. Sci Rep.

[24] Suzuki S, Horinouchi T, Furusawa C (2014). Prediction of antibiotic resistance by gene expression profiles. Nat Commun.

[25] Baquero F, Alvarez-Ortega C, Martinez JL (2009). Ecology and evolution of antibiotic resistance. Environ Microbiol Rep.

[26] Le KY, Park MD, Otto M (2018). Immune Evasion Mechanisms of *Staphylococcus epidermidis* Biofilm Infection. Front Microbiol.

[27] Hall CW, Mah TF (2017). Molecular mechanisms of biofilm-based antibiotic resistance and tolerance in pathogenic bacteria. FEMS Microbiol Rev.

[28] de Silva GD, Justice A, Wilkinson AR, Buttery J, Herbert M, Day NP (2001). Genetic population structure of coagulase-negative staphylococci associated with carriage and disease in preterm infants. Clin Infect Dis.

[29] Foka A, Chini V, Petinaki E, Kolonitsiou F, Anastassiou ED, Dimitracopoulos G (2006). Clonality of slime-producing methicillin-resistant coagulase-negative staphylococci disseminated in the neonatal intensive care unit of a university hospital. Clin Microbiol Infect.

[30] Krediet TG, Mascini EM, van Rooij E, Vlooswijk J, Paauw A, Gerards LJ (2004). Molecular epidemiology of coagulase-negative staphylococci causing sepsis in a neonatal intensive care unit over an 11-year period. J Clin Microbiol.

[31] Widerström M, Monsen T, Karlsson C, Wiström J (2006). Molecular epidemiology of meticillin-resistant coagulase-negative staphylococci in a Swedish county hospital: evidence of intra-and interhospital clonal spread. J Hosp Infect.

[32] Widerström M, Wiström J, Ek E, Edebro H, Monsen T (2011). Near absence of methicillin-resistance and pronounced genetic diversity among Staphylococcus epidermidis isolated from healthy persons in northern Sweden. APMIS.

